# Delayed PCI 12 Hours after the Onset of Symptoms Is Associated with Improved Outcomes for Patients with ST-Segment Elevation Myocardial Infarction: A Real-World Study

**DOI:** 10.1155/2019/2387929

**Published:** 2019-06-18

**Authors:** Wen-Juan Xiu, Hai-Tao Yang, Ying-Ying Zheng, Yi-Tong Ma, Xiang Xie

**Affiliations:** Heart Center, First Affiliated Hospital of Xinjiang Medical University, Urumqi, Xinjiang 830011, China

## Abstract

**Background:**

Primary percutaneous coronary intervention (PPCI) plays a pivotal role in the treatment of ST-segment elevation myocardial infarction (STEMI). However, it remains controversial whether PCI delayed beyond the recommended time window of 12 h after the onset of symptoms is applicable to STEMI.

**Objective:**

The acute myocardial infarction (AMI) registration study in Xinjiang, China, is a real-world clinical trial (retrospective cohort study) that includes hospitalized patients. The purpose of this study was to compare delayed PCI and medication therapy beyond the recommended time window of 12 h after the onset of symptoms on the outcomes of STEMI patients.

**Methods and Results:**

From May 2012 to December 2015, a total of 1072 STEMI patients received delayed PCI (n=594) or standard medication therapy (MT) (n=478) more than 12 h after the onset of symptoms. The number of all-cause deaths in the delayed PCI group and that in the MT group were 55 (9.3%) and 138 (28.9%), respectively, and a significant difference between the groups was indicated for this variable (P<0.001). The number of cardiac deaths in the delayed PCI group and that in the medication therapy group were 47 (7.9%) and 120 (25.1%), respectively, and a significant difference between the groups was indicated for this variable (P<0.001). We also found that the MACE incidence in the delayed PCI group was significantly higher than it was in the MT group (32.2% versus 43.5%, P<0.001). Propensity score matching (PSM) analyses remained significant differences between the delayed PCI group and the MT group, respectively, in all-cause deaths (9.3% versus 25.8%, P<0.001) and cardiac death (8.7% versus 21.6%, P<0.001).

**Conclusion:**

Compared to medication therapy, PCI for STEMI delayed beyond 12 h after the onset of symptoms can better reduce mortality and the incidence of MACEs.

**Trial Registration:**

This study is registered with the following: Trial Registration: clinicaltrials.gov; Identifier: NCT02737956.

## 1. Introduction

Primary percutaneous coronary intervention (PPCI) is an important treatment for ST-segment elevation myocardial infarction (STEMI) [1, 2]. Early myocardial reperfusion is the main target of treatment for acute myocardial infarction (AMI). Existing guidelines have already emphasized the importance of reperfusion of the infarct-related artery (IRA) in this treatment [3]. After AMI, salvaging the myocardium at the earliest possible time is critical. PPCI can open up the IRA, reducing the infarct area and residual stenosis, preserving and improving left ventricular function, and preventing reocclusion. Reducing the time delay of salvaging the myocardium is the primary concern in reperfusion therapy for STEMI. The time from the first medical contact (FMC) to PCI should be reduced, preferably to less than 90 min (I, A). The time from FMC to discharge should also be reduced to lower the risk of in-hospital mortality. ACC/AHA STEMI guidelines recommend performing PPCI within 90 min of FMC (class 1 recommendation) and performing treatment within 120 min of complete ischemia [4, 5]. The European Society of Cardiology (ESC) guidelines also make a similar recommendation for AMI, which is to shorten the time from the onset of STEMI to PCI [6, 7]. In clinical practice, PCI is usually performed within 12 h following STEMI. However, in real-world situations, many patients go to the hospital beyond the 12 h window of the onset of chest pain. Thus, it is disputed whether delayed PCI can deliver the desired outcome. Some studies have shown that standard or optimized medication therapy is better than delayed PCI [8, 9], while others argue for the benefits of delayed PCI for remedy reperfusion therapy [10–16]. The accepted timing of PCI ranges from 2 days to 60 days after the onset of STEMI, though the long-term prognosis of each differs and some contradictory conclusions have been drawn [17, 18].

The 2016 Chinese guidelines for PCI consider performing PCI on those with clinical ischemia and (or) evidence of ischemia within 12-48 h after the onset of symptoms to be feasible (class of recommendation IIa, level of evidence B) [19]. However, there is a lack of evidence of whether PPCI should be performed beyond 12 h after the onset symptoms in those without explicit evidence of ischemia. This study compared the clinical outcomes of STEMI patients who received delayed PCI and STEMI patients who received medication therapy.

## 2. Methods

### 2.1. Study Design

The registration study of AMI in Xinjiang, China, is a multicenter clinical trial initiated by the First Affiliated Hospital of Xinjiang Medical University without any sponsorship from enterprises. Consecutive patients with STEMI who were hospitalized at the First Affiliated Hospital of Xinjiang Medical University from May 2012 to December 2015 were recruited. The research protocol was approved by the ethics committee or review committee of the First Affiliated Hospital of Xinjiang Medical University. Because the study was a retrospective cohort study based on real-world situations, there was no need to obtain informed consent from the patients.

### 2.2. Subjects

A total of 1072 STEMI patients who received treatment beyond the 12 h time window at the First Affiliated Hospital of Xinjiang Medical University were included in this study. The inclusion criteria were as follows: (1) Continuous chest pain for over 30 min without remission; (2) ST-elevation on ECG (without left ventricular hypertrophy or a left bundle branch block): J-point elevation in two adjacent leads, the cutoff being ≥0.2 mV for men and ≥0.15 mV for women, and (or) a cutoff of ≥0.1 mV in the other leads; (3) appearance of a pathological Q-wave on ECG; (4) New radiologic finding of a loss of viable myocardium or local heart wall motion abnormality; (5) beyond the 12 h window after the onset of chest pain. The patients were excluded if they met one or more of the following criteria: (1) tumor, liver, and kidney insufficiency; (2) life expectancy less than 6 months; (3) LVEF<30%; (4) previous thrombolysis recipient.

### 2.3. PCI Group

The patients were given oxygen therapy, analgesics, and standard medication therapy. The medication therapy consisted of 75 mg oral clopidogrel once daily, 100 mg aspirin once daily, and low-molecular-weight heparin and/or glycoprotein IIb/IIIa antagonists. The patients were given the preoperative load (150 mg clopidogrel) on the night before surgery. All STEMI patients without contraindications were given statins, *β*-receptor inhibitors, ACEI, or ARB as preventive therapy. The patients received coronary angiography (CAG) to determine the necessity of stent implantation in IRA. Non-IRA was also treated appropriately. Occlusion of IRA was defined as 100% stenosis with a TIMI grade of 0 or 1.

### 2.4. Medication Therapy Group

The patients were given oxygen therapy, analgesics, and standard medication. The standard medication was the same as that used in the PCI group.

### 2.5. Clinical Endpoints and Follow-Up

The primary endpoints were all-cause death; the secondary endpoint was MACE, which consisted of cardiac death, revascularization, reinfarction, stroke, and rehospitalization for heart failure. We also recorded bleeding events. All patients were followed up by telephone or mail. MACEs were observed, and every endpoint event was recorded. A single patient might undergo one or several MACEs, but all of them were recorded. The median follow-up was 26 months (0-55 months).

### 2.6. Data Analysis

The baseline data and continuous variables of CAG that showed a normal distribution were presented as the mean ± standard deviation (SD). The measurement data were analyzed by an independent samples t-test, and the count data were analyzed by the chi-square test. Kaplan-Meier survival curves were drawn for univariate survival analysis. The survival rates between the subgroups were examined by the log-rank test. Multivariate survival analysis was conducted using the Cox regression model.

To reduce selection bias and potential confounding factors, propensity score matching (PSM) was adopted for the adjustment of baseline clinical characteristics. After 1:1 matching, 666 patients were selected for the final analysis, including 333 AMI patients who received delayed PCI and 333 AMI patients who received medication therapy. The following variables were considered: age, gender, heart rate, smoking status, and Killip grade. The chi-square test and independent samples t-test were reimplemented during matching to test for the differences in the variables between the two groups after matching. All data were analyzed statistically using SPSS 24.0. P<0.05 was considered significant.

## 3. Results

A total of 1072 STEMI patients hospitalized from May 2012 to December 2015 were included, with 594 patients in the delayed PCI group (12 h<t<28 d) and 478 patients in the medication therapy group.

The patients were aged 24-95 years (58.49±12.81 years), and there were 877 (81.8%) men and 195 (18.2%) women. There were 259 patients with diabetes, accounting for 24.2% of the total patient population; 330 patients with hypertension, accounting for 31% of the total patient population; and 252 patients with hyperlipidemia, accounting for 23.5% of the total patient population. According to the Killip classification, there were 23 patients with grade 1 cardiac function, accounting for 2.1% of the total patient population; 817 patients with grade 2 cardiac function, accounting for 76.2% of the total patient population; and 232 patients with grade 3-4 cardiac function, accounting for 21.7% of the total patient population.

### 3.1. Comparison of Baseline Data

Compared with those in the delayed PCI group, the patients in the medication therapy group were older (P<0.001) and had a higher heart rate (P<0.001) and worse cardiac function (P<0.001), and there were more men in this group than there were in the delayed PCI group (P=0.004). The two groups showed a significant difference in smoking status as a high-risk factor (P=0.003). However, there was no significant difference in total cholesterol, low-density lipoprotein cholesterol or the prevalence of high-risk factors for coronary heart disease, such as diabetes, hypertension, and hyperlipidemia (all P>0.05) (Table 1).

### 3.2. Primary Endpoints

The number of all-cause deaths in the delayed PCI group and that in the medication therapy group were 55 (9.3%) and 138 (28.9%), respectively, which indicates a significant difference (P<0.001). The incidence of all-cause death was much higher in the medication therapy group than it was in the delayed PCI group (Table 2).

### 3.3. All-Cause Death

**Figure 1:**
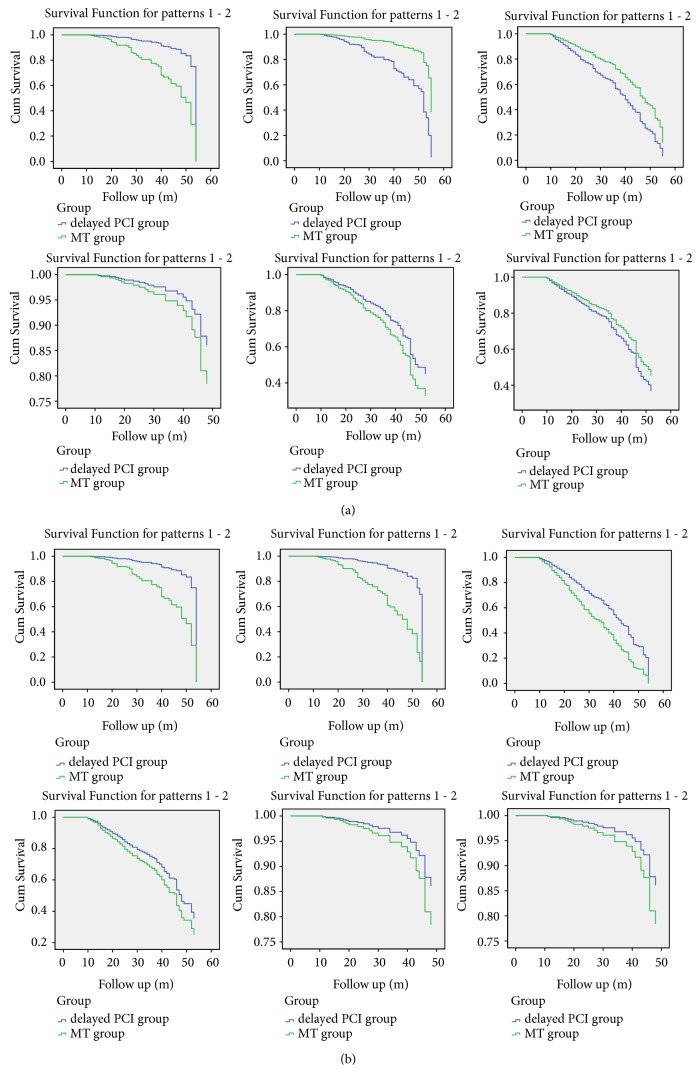
Kaplan-Meier curves for event (ACM, CM, MACEs, Rehospitalization for HF, Revascularization, and Stroke, respectively) free survival according to therapy for STEMI after 12 hours of the onset of symptoms. (a) Before matching; (b) after matching.

Kaplan-Meier survival curves and the log-rank test (pooled over strata) revealed a significant difference in the incidence of all-cause death between the two groups (P < 0.001) (Figure 1(a)).

Potential influence of factors of survival was analyzed using the multivariate model. After eliminating the interactions between the factors influencing all-cause death, a Cox regression model indicated that the treatment regimen was an independent prognostic factor in STEMI (P < 0.001). Compared with the prognosis in the medication therapy group, the prognosis in the delayed PCI group was considerably improved. The risk of all-cause death was lower after delayed PCI than that after medication therapy (HR=0.262, 95% CI: 0.164-0.417, P<0.001, Table 3).

### 3.4. Secondary Endpoints

The numbers of patients presenting with MACE were 191 (32.2%) and 208 (43.5%) in the delayed PCI group and medication therapy group, respectively, and a significant difference between the groups was shown (P<0.001). The number of cardiac deaths in the delayed PCI group and that in the medication therapy group were 47 (7.9%) and 120 (25.1%), respectively, and a significant difference between the groups was shown (P<0.001). The incidence of MACE and cardiac death was much higher in the medication therapy group than in the delayed PCI group (Table 2).

### 3.5. MACE

Kaplan-Meier survival curves and the log-rank test (pooled over strata) found a significant difference in the incidence of MACE between the two groups (P=0.001) (Figure 1(a)).

After eliminating the interaction between the influencing factors for MACE, a Cox regression model found that the treatment regimen was an independent influencing factor for MACE in STEMI patients. Compared with medication therapy, delayed PCI (12 h<t<28 d) greatly improved the prognosis. The risk of MACE in the patients who received delayed PCI was lower than that in the patients who received medication therapy (HR=0.528, 95% CI: 0.406-0.686, P<0.001, Table 3).

### 3.6. Cardiac Death

Kaplan-Meier survival curves and the log-rank test (pooled over strata) revealed a significant difference in the incidence of cardiac death between the two groups (P<0.001) (Figure 1(a)).

Potential influencing factors of survival were analyzed using the multivariate model. After eliminating the interactions between the influencing factors of cardiac death, a Cox regression model indicated that the treatment regimen was an independent prognostic factor in STEMI (P<0.001). Compared with the prognosis in the medication therapy group, the prognosis in the delayed PCI group was considerably improved. The risk of cardiac death was lower after delayed PCI than after medication therapy (HR=0.286, 95% CI: 0.173-0.474, P<0.001) (Table 3).

### 3.7. PSM Analysis

PSM analysis was used for adjustment of the baseline clinical characteristics. After 1:1 matching, 666 patients were selected for the final analysis, including 333 AMI patients who received delayed PCI and 333 AMI patients who received medication therapy.

The patients were aged 24-94 years (58.49±12.81 years), and there were 539 (80.9%) men and 127 (19.1 %) women. There were 168 patients with diabetes, accounting for 25.2% of the total patient population; 192 patients with hypertension, accounting for 28.2% of the total patient population; and 153 patients with hyperlipidemia, accounting for 22.9% of the total patient population. According to Killip classification, there were 13 patients with grade 1 cardiac function, accounting for 2% of the total patient population; 514 patients with grade 2 cardiac function, accounting for 76.2% of the total patient population; and 232 patients with grade 3-4 cardiac function, accounting for 20.9% of the total patient population.

The number of all-cause deaths in the PCI group and that in the medication therapy group were 31 (9.3%) and 86 (25.8%), respectively (P<0.001). The incidence of MACE was much higher in the medication therapy group than in the delayed PCI group (43.5 versus 38.4%, P=0.092). The number of cardiac deaths in the delayed PCI group and that in the medication therapy group were 29 (8.7%) and 72 (21.6%), respectively (P<0.001) (Table 4).

Kaplan-Meier survival curves and the log-rank test (pooled over strata) indicated a significant difference in the incidence of all-cause death, MACE, and cardiac deaths between the two groups (P<0.001, P=0.001, and P<0.001, respectively) (Figure 1(b)). These results indicated that, compared to medication therapy, delayed PCI significantly improved the prognosis of STEMI patients.

## 4. Discussion

Our results indicated that, beyond a 12 h time window, delayed PCI was superior to standard medication therapy in improving the prognosis of STEMI patients.

The TOSCA-2 trial [20], which was published in 2006, showed that the patency of IRA 1 year after PCI was much higher than that after medication therapy. However, the two groups showed no significant difference in LVEF (P=0.47). It was found that the long-term patency of the IRA being reopened in the recovery period of AMI was satisfactory. In 2008, Abbate A [21] conducted a meta-analysis of 10 randomized controlled trials, including the OAT study, the SWISSI II study, and the BRAVE-2 study, comparing the efficacy of PCI and medication therapy following STEMI (12 h-30 d). The results showed that mortality was significantly improved in the PCI group compared to that in the medication therapy group (OR=0.49, 95% CI: 0.2-0.94, P=0.03). PCI also outperformed medication therapy in cardiac remodeling (P=0.009). This meta-analysis showed that delayed reopening of the IRA (12 h-60 d) could still improve cardiac function and survival. Degeare VS [22] believed that following AMI, IRA could prevent left ventricular remodeling, keeping the reopened vessels unobstructed and helping to achieve a TIMI grade 3 flow. Thus, PCI plays a crucial role in opening up the IRA and keeping it unobstructed, in preventing left ventricular remodeling and in protecting cardiac function. In our study, a multivariate Cox regression model found that delayed PCI was superior to simple medication therapy in terms of reducing cardiac death, all-cause death, and MACE (HR=0.286, 95% CI: 0.173-0.474, P < 0.001; HR=0.262, 95% CI: 0.164-0.417, P < 0.001; HR=0.723, 95% CI: 0.563-0.929, P=0.011). Our results are consistent with the results of the meta-analysis observed by Abbate et al. [21]. Multivariate Cox regression analysis of the baseline clinical characteristics was performed to identify the influencing factors of cardiac death, all-cause death and MACE. The analysis showed that baseline clinical characteristics were not independent influencing factors for prognosis in STEMI patients. This result implied that there was potentially the presence of selection bias.

Our study was a retrospective trial. Delayed PCI was performed only for those surviving in the acute phase of AMI, and the critically ill patients who died in the acute phase were excluded. Patients receiving simple medication therapy were usually those who were older, had poor cardiac function and had underlying diseases. Therefore, patients receiving PCI tended to be those who were at a lower risk for complications compared with the patients receiving medication therapy. This was a major source of selection bias.

To reduce selection bias and potential confounding factors, PSM analysis was adopted for the adjustment of the baseline clinical characteristics. After 1:1 matching, 666 patients were selected for the final analysis, including 333 AMI patients who received delayed PCI and 333 AMI patients who received medication therapy.

Multivariate Cox regression analysis indicated that delayed PCI was superior to medication therapy in reducing both cardiac death and all-cause death (HR=0.221. 95% CI: 0.123-0.397, P < 0.001; HR=0.193, 95% CI: 0.112-0.334, P < 0.001). This result is in agreement with the results from the meta-analysis conducted by Abbate A. Our research also found that the incidence of MACE did not differ significantly between the delayed PCI group and medication therapy group (P=0.066). However, compared with medication therapy, delayed PCI greatly improved the prognosis of these patients (12 h<t<28 d), and the risk of MACE was reduced in the delayed PCI group more so than in the medication therapy group (HR=0.766, 95% CI: 0.566-1.018). Multivariate Cox regression analysis was performed on the baseline clinical characteristics to assess the influencing factors of cardiac death, all-cause death, and MACE. Killip grade was an independent influencing factor of cardiac death and all-cause death in STEMI patients. This result may be related to the rematching of patients and to the selection of the baseline clinical characteristics.

Time is the most important factor in the reopening of IRA. However, none of the patients in our study received early reperfusion therapy. The reasons for this may include the following: (1) delayed diagnosis: the patients were usually not fully aware of the danger of AMI, and less specialized hospitals lacked experience in making an early diagnosis of AMI; (2) fear of the risk that PCI posed in elderly patients: many elderly patients did not receive PCI because of their advanced age, which reflects concern with the safety of PCI the physicians. In fact, for elderly AMI patients, the risk of death is high with or without PCI; (3) the patients and their relatives were hesitant in making a treatment decision, which led to a delayed intervention.

It will be of a high significance to assess the benefits of late reperfusion for AMI beyond a 12 h window but not exceeding 2-60 d. 2017 ESC STEMI guidelines give a class IIaB recommendation for primary PCI (PPCI) beyond 12 hrs [22]. 2018 myocardial revascularization ESC/EACTS guidelines give a class IIaB recommendation for a routine primary PCI strategy that indicates that primary PCI should be considered in patients presenting late (12–48 h) after the onset of symptoms [23]. However, selective PCI cannot salvage the dead cardiomyocytes in the acute phase of AMI but can rather improve cardiac function and prognosis by reducing myocardial remodeling and promoting functional recovery of the surviving myocardium in the infarct region. Selective PCI may improve prognosis through several pathways: (1) restoring antegrade flow in the vessels supplying the infarction region, thus salvaging the hibernating myocardium, and alleviating reversible myocardial damage and promoting its functional recovery; (2) improving scar repair of the infarct tissues, reducing myocardial remodeling in the noninfarct region, and preventing the extension of infarct region and ventricular dilation; (3) reducing electrical instability of the myocardium and malignant ventricular arrhythmia; (4) increasing collateral circulation near the infarct and alleviating myocardial ischemia.

## 5. Limitations

Our study has the following limitations. First, for a retrospective study design, the research findings were closely related to the accuracy and integrity of medical records. Second, the patients were not selected by random sampling but were all patients at the department of cardiology at the First Affiliated Hospital of Xinjiang Medical University, which may give rise to selection bias. Finally, several variables, such as the interval from the onset of symptoms to diagnosis and the ejection fraction, were not included in the PSM analysis. Furthermore, there may be some residual unknown confounding factors in our analysis despite PSM being performed.

## 6. Conclusions

PCI delayed beyond a 12 h window for STEMI patients was conducive to reducing long-term mortality.

## Figures and Tables

**Table 1:** Clinical baseline characteristics and angiographic characteristics of included patients before and after PSM.

*Characteristics*	Before Matching			After Matching		
	
	Delayed PCI (n=594)	MT (n=478)	P value	Delayed PCI (n=333)	MT (n=333)	P vale
Age, yrs	56.63±11.15	60.79±14.28	<0.001	57.34±11.07	58.73±14.51	0.166
Male, n (%)	504 (84.8)	373 (78)	0.004	276 (82.9)	263 (79)	0.20
SBP (mmHg)	119.61±17.64	121.34±22.19	0.189	119.46±17.79	122.12±22.49	0.082
Diabetes, n (%)	149 (25.1)	110 (23)	0.431	78 (23.4)	90 (27)	0.284
Hypertension, n (%)	181 (30.5)	149 (31.2)	0.805	98 (29.4)	94 (28.2)	0.732
Hyperlipidemia, n (%)	142 (239)	110 (23)	0.732	71 (21.3)	82 (24.6)	0.311
Current smoker, n (%)	260 (43.9)	168 (35.1)	0.003	152 (45.6)	140 (42)	0.349
LDL-C (mmol/L)	2.48±0.89	2.44±0.91	0.472	2.54±0.93	2.42±0.92	0.093
TC (mmol/L)	3.84±1.11	3.86±1.12	0.803	3.88±1.13	3.81±1.14	0.466
Killip's class			<0.001			0.819
1	17 (2.9)	6 (1.3)		7 (2.1)	6 (1.8)	
2	508 (85.5)	309 (64.6)		258 (77.5)	256 (76.9)	
3	55 (9.3)	100 (20.9)		55 (16.5)	53 (15.9)	
4	14 (2.4)	63 (13.2)		13 (3.9)	18 (5.4)	
***IRA, n (***%**)**			NA			NA
Left main	0	NA		0	NA	
LAD	385 (68.0)	NA		224 (67.3)	NA	
LCX	51 (8.5)	NA		26 (7.8)	NA	
RC	158 (26.6)	NA		83 (24.9)	NA	
***TIMI flow grade n (***%**)**			NA			NA
*0*	380 (64.0)	NA		212 (63.7)	NA	
*1*	185 (31.1)	NA		108 (32.4)	NA	
*2*	29 (4.9)	NA		13 (3.9)	NA	
ACEI/ARB, ***n (***%**)**	307 (51.7)	266 (55.6)	0.316	180 (54.1)	191 (57.4)	0.521
DAP, ***n (***%**)**	524 (88.2)	416 (87)	0.557	292 (87.7)	300 (90.1)	0.324
Anticoagulation, ***n (***%**)**	282 (47.5)	210 (43.9)	0.277	163 (48.9)	143 (42.9)	0.292
*β*-Blocker	409 (68.9)	311 (65.1)	0.101	235 (70.6)	227 (68.2)	0.361
Calcium Blocker	123 (20.7)	79 (16.5)	0.082	64 (19.2)	54 (16.2)	0.310
Statins	458 (77.1)	365 (77.3)	0.670	250 (75.1)	258 (77.5)	0.499

Note: TC: total cholesterol; LDL-C: low density lipoprotein cholesterol; IRA: infarct-related artery; LAD: left anterior descending; LCX: left circumflex; RC: right coronary; DAP: double antiplatelet.

**Table 2:** Clinical events during follow-up.

Events	Delayed PCI(n=594)	MT (n=478)	95% CI	P value
*Primary endpoints, n (*%)				
Cardiac death	47 (7.9)	120 (25.1)	0.173-0.403	<0.001
All-cause mortality	55(9.3)	138 (28.9)	0.170-0.365	<0.001
*Secondary endpoints, n (*%)				
MACEs	220(37.0)	226(47.3)	0.626-0.958	<0.001
Rehospitalization for HF	142(23.9)	86(18.0)	0.604-1.038	0.092
Revascularization	25(4.2)	16(3.3)	0.391-1.394	0.350
Stroke	6(1.0)	4(0.8)	0.201-2.625	0.626
Blooding events	14(2.4)	8(1.7)	0.338-1.959	0.646

**Table 3:** Cox proportional hazards analysis of cardiac death, ACM, and MACEs.

Characteristics	HR (95% CI)	P values
*Cardiac death*		
Age, yrs	1.003(0.983-1.024)	0.741
Gender	1.477(0.840-2.596)	0.175
LDL-C	1.010(0.723-1.411)	0.955
TC	0.922(0.702-1.210)	0.558
Current smoker	1.345(0.779-2.321)	0.288
Killip's class	1.125(0.777-1.630)	0.532
Treatment	0.286(0.173-0.474)	<0.001

*ACM *		
Age, yrs	1.007(0.988-1.025)	0.489
Gender	1.525(0.918-2.531)	0.103
LDL-C	1.086(0.824-1.431)	0.559
TC	0.902(0.712-1.143)	0.304
Current smoker	1.303(0.786-2.162)	0.305
Killip's class	1.239(0.889-1.733)	0.209
Treatment	0.262(0.164-0.417)	<0.001

*MACEs*		
Age, yrs	0.995(0.985-1.009)	0.319
Gender	0.976(0690-1.379)	0.889
LDL-C	0.936(0.785-1.116)	0.46
TC	1.048(0.910-1.207)	0.518
Current smoke	1.011(0.786-1.301)	0.93
Killip's class	0.987(0.793-1.229)	0.91
Treatment	0.723(0.563-0.929)	0.011

**Table 4:** Clinical events during follow-up after PSM analysis.

Events	PCI (n=333)	MT (n=333)	95% CI	P Value
*Primary endpoints*				
Cardiac death, n (%)	29 (8.7)	72 (21.6)	0.136-0.403	<0.001
All -cause mortality, n (%)	31(9.3)	86 (25.8)	0.121-0.333	<0.001
*Secondary endpoints * **(**%**)**				
MACEs	128(38.4)	145(43.5)	0.545-0.927	<0.001
Rehospitalization for HF	81(24.3)	60(18)	0.514-1.014	0.060
Revascularization	14(4.2)	11(3.3)	0.275-1.375	0.237
Stroke	4(1.2)	2(0.6)	0.169-5.451	0.963
Blooding events	11(3.3)	6(1.8)	0.351-2.638	0.94

## Data Availability

Due to confidentiality policies, the data used in this study will not be shared.

## Abbreviations

PPCI: Primary percutaneous coronary intervention
STEMI: ST-segment elevation myocardial infarction
PSM: Propensity score matching
AMI: Acute myocardial infarction
CAG: Coronary angiography
LVEF: Left ventricular ejection fraction
IRA: Infarct-related artery
ACM: All-cause mortality
MACE: Major adverse cardiovascular events.
